# Facilitating sleep initiation in children with ADHD and sleep problems: a qualitative experience-based study

**DOI:** 10.1186/s12887-025-05964-3

**Published:** 2025-08-07

**Authors:** Mirjam Alvstrand, Maria Lönn, Petra Svedberg, Nygren, Ingrid Larsson

**Affiliations:** 1 Department of Health and Care, School of Health and Welfare, Halmstad University, Halmstad, Sweden; 2 Psychiatry Halland, Region Halland, Halmstad, Sweden

**Keywords:** Attention deficit hyperactivity disorder (ADHD), Children, Interviews, Qualitative content analysis, Sleep initiation

## Abstract

**Background:**

Children with Attention Deficit Hyperactivity Disorder (ADHD) often experience significant sleep difficulties, which can impair daily functioning and exacerbate ADHD symptoms. Sleep-related challenges, such as difficulties with sleep initiation, not only affect the child but also disrupt family dynamics, thereby impacting general family well-being. Understanding the factors that facilitate better sleep initiation could provide valuable insights to inform the development of targeted interventions to support children with ADHD and associated sleep problems.

**Aim:**

This study aimed to explore the experiences of facilitators supporting sleep initiation among children with ADHD and sleep problems.

**Method:**

An exploratory, inductive qualitative design was used. Individual interviews were conducted with 21 children (11 boys and 10 girls) aged 6–12, diagnosed with ADHD and sleep problems. Data were analyzed through qualitative content analysis, resulting in five subcategories and two main categories.

**Results:**

The findings revealed that children with ADHD and sleep difficulties identified both behavioral and environmental factors that facilitate sleep initiation. Behavioral factors included engaging in physical exercise, regulating digital device use, and maintaining a balance between activity and rest. Environmental facilitators included a calming sleeping environment and the maintenance of consistent evening routines. The children highlighted the importance of individualized approaches to the integration of these strategies, to effectively support sleep initiation.

**Conclusions:**

This study highlights the relevance of both behavioral and environmental aspects in supporting sleep initiation among children with ADHD. These exploratory findings may inform future research and practice by emphasizing the value of integrating children’s own perspectives and preferences when designing supportive strategies.

## Introduction

Sleep is essential for children’s overall health, well-being, and daily functioning. It serves as a foundation for their physical, mental, and emotional development [1, 2]. Adequate sleep, which includes both sufficient duration and high quality, plays a vital role in supporting attention, regulating behavior, enhancing cognitive abilities, and promoting overall health [2]. For children with neurodevelopmental disorders, such as attention deficit hyperactivity disorder (ADHD), sleep is even more critical [3, 4]. Getting adequate sleep is key to their daily functioning, academic achievement, and successful transition into adulthood [3, 5].

Children with ADHD are particularly vulnerable to sleep problems, with up to 70% experiencing poor sleep quality and frequent disturbances [6, 7], compared to just 20–30% of children without ADHD [8]. These sleep difficulties significantly impact various areas of development, including behavioral regulation, cognitive processing, and executive functioning [9]. Common sleep-related challenges include resistance to bedtime, delayed sleep initiation, poor sleep quality, sleep anxiety, frequent awakenings [10] and restless sleep [11].

When sleep is disrupted, the consequences extend beyond the child, affecting family dynamics and overall household well-being [10, 12, 13]. Parenting practices and sleep hygiene play key roles in mitigating these challenges. Consistent parenting and good sleep hygiene are associated with improved sleep outcomes, such as reduced sleep-related anxiety, less daytime fatigue, and fewer nighttime disruptions. Sleep hygiene includes evening and bedtime routines and optimization of the sleep environment [14]. However, adolescents have reflected on the challenges of implementing it during childhood [15]. Additionally, inconsistent sleep patterns between weekdays and weekends [15–17], and screen use before bedtime are significant disturbing factors that may exacerbate sleep initiation difficulties, lead to night awakenings and intensify ADHD symptoms [18, 19].

Inadequate sleep in children with ADHD can have long-term consequences on behavior, daily functioning [20], learning, and emotional regulation [21]. Furthermore, sleep disruptions can negatively affect concentration, academic performance, emotional stability [10], and social interactions, potentially leading to issues such as social isolation, low self-esteem, and underachievement [22]. Altogether, insufficient sleep may strain family relationships and further exacerbate the challenges of managing ADHD symptoms [13].

Despite the critical importance of sleep, existing research on children with ADHD and sleep problems is predominantly quantitative and often relies on parental reports [9, 14, 18, 19], and parents’ experiences [23, 24], with limited attention to the children’s own experiences. This approach may oversimplify sleep initiation, a key aspect of children’s sleep health, which, if disrupted, can affect behavior, attention, and cognitive function, common challenges for children with ADHD. Studies have explored adolescents’ perceptions of sleep [15, 25–27] as well as how children and adolescents with ADHD manage their symptoms [22]. However, there is a lack of research specifically focusing on the subjective experiences of children with ADHD regarding sleep problems and difficulties with sleep initiation [10].

While the importance of sleep for children with ADHD is well-established, little is known about how they themselves experience and manage sleep initiation. Gaining a deeper understanding of their experiences can provide valuable insights, to inform the development of more effective interventions, ultimately improving sleep outcomes and overall functioning. In contrast to previous studies relying on parental reports and experiences [9, 14, 18, 19, 23, 24], this study brings attention to the children`s valuable perspective. Therefore, the aim of this study was to explore the experiences of facilitators that support sleep initiation among children with ADHD and sleep problems.

## Materials and methods

### Study design

This study has an explorative design, applying an inductive, qualitative content analysis approach to describe and interpret the manifest content of the interviews. The purpose of this qualitative content analysis is to identify similarities and differences in the children’s descriptions and to systematically categorise the content, staying close to the data [28]. To ensure trustworthiness, the study is reported in accordance with the Consolidated Criteria for Reporting Qualitative Research (COREQ) 32-item checklist [29].

### Participants

This study is part of a larger research project– a sleep intervention with weighted blankets conducted between January 2020 and June 2022, in collaboration between Halmstad University and the ADHD unit at a Child and Adolescent Mental Health Service in Sweden [30]. The inclusion criteria comprised children with a recent diagnosis of uncomplicated DSM-5 ADHD, i.e., without significant comorbidities (including inattentive, hyperactive, and combined subtypes), who were either on stable pharmacological treatment or no medication, and had sleep problems confirmed by three selected questions from the Child’s Sleep Habits Questionnaire (CSHQ). These questions addressed whether the child frequently or occasionally experienced problems falling asleep within 20 min of going to bed, slept too little, or woke up several times per night [31]. An exclusion criterion was that both parents and children needed to understand (written and spoken) the Swedish language. The age of the participating children in this study was 6–12 (median age 9.5 years), 11 boys, and 10 girls.(Table 1).

**Table 1:** Description of the participants

Age	6–8 years	9–10 years	11–12 years	All
Number of participants (n)	6	9	6	21
Gender; boys/girls	2/4	5/4	3/3	10/11
ADHD subtype; Inattentive/hyperactive/combined (n)	1/1/4	2/0/7	3/0//3	6/1/14
Comorbidities Oppositional defiant disorder (ODD) (n) Language disorder (n)	1		1 2	2 2
CGAS median (min-max)	53 (49–55)	53 (49–55)	52 (42–56)	53 (42–56)
CSHQ Total score, median (min-max)	51 (38–63)	48 (44–61)	49 (40–58)	48 (38–63
ISI Total score, median (min-max)	8 (4–17)	12 (7–18)	14 (3–17)	10 3–18
Stimulant medication (n)	1	4	2	7
Sleep medication (melatonin) (n)		2		2
Alternates between two homes (n)	0	3	2	5
Number of siblings (0/1/2) (n)	1/1/4	1/5/3	0/5/1	2/11/8
Single parent (n)	2	2	2	6
Parents’ education; Upper secondary/university (n)	2/4	3/6	6/0	11/10
Parents’ employment Full‑time/Part‑time/Student/Parental or sick leave (n)	1/4/1/0	6/1/0/2	5/0/1/0	12/5/2/2
Parent’s origin Native born/Non‑native born (n)	6/0	7/2	6/0	19/2

Children’s Global Assessment Scale (CGAS) (scores range from 1 to 100, worse to best) [32]; Child’s Sleep Habits Questionnaire (CSHQ) Total Score (scores range from 33 to 99, best to worse) [31]; Insomnia Severity Index (ISI) (scores range from 0 to 28, best to worse) [33].

### Data collection

A total of 21 individual interviews with the children were conducted between May 2020 and September 2021 by (ML, PS, and KA). Twelve children were interviewed face-to-face at Halmstad University (May-September 2020) in a separate room, with a parent present in an adjacent room. Due to restrictions imposed during the COVID-19 pandemic, nine children were interviewed in their home environment through the digital platform Zoom (February 2021-September 2021).

A semi-structured interview guide was used that included questions about the children’s experience related to sleep and circumstances around sleep, such as “how do you usually sleep?”, “in what way can it be difficult to fall asleep?”, and “what is important for you to fall asleep?” and “what is important to you in order for you to sleep well?”. The children were encouraged to elaborate through in-depth questions such as ‘Can you tell me more?’ or ‘Can you give examples?”. A pilot interview was conducted in order to test the interview guide. No changes to the interview guide were deemed necessary, and the pilot interview was included in this study. The interviews lasted between 13 and 62 minutes (median: 39 minutes), and in total amounted to 13 hours and 44 minutes. Data saturation was reached after the 16th interview, as no new relevant information emerged. This was confirmed by five additional interviews to ensure the categories captured all meaningful aspects of the children’s experiences. All interviews were audio recorded and transcribed verbatim.

Recognizing that interviewing children, especially those with ADHD, can be challenging, accommodations were made to facilitate honest and open responses. Interviewers used simplified language, allowed breaks whenever needed, and encouraged the children to ask questions if something was unclear. Material (a night and day timeline and speech bubbles about their sleep) was used during the face-to-face interviews. This material helped the children visualize their experiences in a concrete and accessible way.

### Data analysis

An inductive, qualitative content analysis was used to analyze the interviews [28]. Initially, all transcripts were read and reread to understand the children’s experiences of sleep initiation facilitators and to obtain a sense of the whole. Sections of the transcripts relevant to the study’s aim were identified and extracted, while preserving the context and the original meaning. These sections, referred to as meaning units, contained expressions related to children’s everyday life experiences. The identified meaning units were then condensed to shorten the text while retaining its core content. In the next step, the condensed meaning units were abstracted and coded. The codes captured the essence of the experiences described and allowed for comparisons across interviews. The codes were then sorted and compared based on similarities and differences, and grouped into five subcategories. The five subcategories were grouped into two categories, based on similarities and differences. For example, the subcategories *performing physical activities*,* using digital devices* and *balancing activities and resting* were merged into the category of *adapting behavioral factors *. This step-wise process of reading, extracting meaning units, condensing, coding, and developing subcategories and categories followed the method outlined by Graneheim, Lindgren, and Lundman [28]. To ground the analysis in the children’s voices and ensure that the findings reflect their lived experiences, 2–4 quotations from different children were included for each subcategory. These quotations illustrate the categories and highlight the diversity of experiences among the children.

Data were analysed by the first (MA) and last authors (IL), and the coding process was regularly discussed. The group, which included a teacher (MA), an occupational therapist (ML), two registered nurses (PS, IL), and a biomedicinal scientist (JN), brought diverse perspectives to the analysis and interpretation of the data. Discrepancies in coding or category formulation were resolved through discussion until consensus was reached. This iterative and collaborative process contributed to the trustworthiness and credibility of the findings. Throughout the analytic process, the researchers’ preunderstandings as professionals and as parents were continuously reflected upon and discussed to ensure that the children’s voices remained central to the interpretation. This reflexive approach contributed to the confirmability and credibility of the analysis.

### Ethical considerations

The study was approved by the Swedish Ethical Review Authority (Sweden, no. 2019–02158) and adhered to the principles outlined in the Declaration of Helsinki [34] as well as the ethical guidelines of the Swedish Research Council [35]. Written informed consent was obtained from all parents and assent was obtained from the children during their initial visit to the Child and Adolescent Mental Health Service (CAMHS) prior to participation. Both parents and children received verbal and written information about the study’s purpose, procedures, and their rights, including the right to withdraw at any time without any consequences for their care. Participation was voluntary, and the children were explicitly informed that they could withdraw at any time. Confidentiality was ensured by restricting access to study materials to authorized research team members only. All data were de-identified and securely stored in accordance with the General Data Protection Regulation (GDPR) [36].

## Results

The findings revealed that children with ADHD and sleep problems described adaptations of both behavioral and environmental factors as important for facilitating sleep initiation. Behavioral adaptations included engaging in physical activities, using digital devices, and balancing activity with rest. Environmental adaptations involved the establishment of a calming sleep environment and consistent evening routines, often structured by parents, to support falling asleep (Table 2).

**Table 2:** The categories and subcategories explore the experiences of facilitators in supporting sleep initiation among children with ADHD and sleep problems

Categories	Adapting behavioral factors	Adapting environmental factors
Subcategories	Performing physical activities	Creating a calming sleep environment
	Using digital devices	
		Establishing consistent evening routines
	Balancing activities with rest	

### Adapting behavioral factors

The children experienced that adapting behavioral factors were essential in facilitating sleep initiation, making falling asleep easier. Engaging in regular physical activities helped them feel naturally tired by evening, while the controlled use of digital devices, such as calming videos or specific games, aided in winding down before bedtime. They also mentioned balancing stimulating activities with periods of relaxation throughout the day, noting that this approach better prepared them for sleep.

#### Performing physical activities

The children described that having routines for engaging in physical activities influenced their ability to fall asleep. However, the specific activities that had the most beneficial impact on their ability to unwind varied individually. The children believed that their level of physical activity during the day directly affected their ability to initiate sleep.

*If I’ve just been lying on the couch all day*,* I find it a bit harder to fall asleep… But if I’ve been up and about*,* staying busy and all that*,* I usually fall asleep pretty quickly.* (Child 10 years)

The children expressed a need for physical activity, preferably outdoors, to become tired in the evening and unwind.

*I have to be outside or do something that makes me tired*,* otherwise I won’t be able to sleep. It’d be harder to fall asleep… Going for a walk or jumping on a trampoline can make me sleep better and become more tired.* (Child 12 years)

Some children felt that the type of physical activity they engaged in made a difference, as certain activities could leave them more energized and thus make it harder to fall asleep. For instance, one child mentioned, *”If I’ve been jumping on a trampoline*,* it’s harder to sleep”* (Child 10 years). The children also noted differences in their activity levels between weekdays and weekends. They tend to be more active on weekdays, while weekends are often more sedentary. *“Some weekends I just sit at home and do nothing”* (Child 12 years). These experiences highlight the importance of individualized physical activity routines, where adjusting the timing of physical activity could support sleep initiation.

#### Using digital devices

The children described having a routine of using digital devices such as mobile phones, tablets, computers, TV or radio before bedtime, but they experienced varying effects. Their activities ranged from listening to music, audiobooks, and stories to watching movies, playing games, and engaging in social media. For some children, this digital time served as a way to unwind and relax. As one child noted, *”I usually get relaxed sometimes when I play (video games).”* (Child 9 years). They found that focusing on the screen helped them tune out disturbing thoughts that could otherwise hinder sleep initiation. Others described that screen time had the opposite effect, making them more alert.

…*when I turn on the iPad*,* I actually become quite awake. That’s why we only have them on weekends*,* because then we can stay up late and sleep in a bit longer.* (Child 8 years)

The children stated that the impact of digital devices on sleep initiation depended on how they were used, including the type of content, duration of use, and screen brightness. Social media was considered particularly stimulating, making it harder to initiate sleep. They suggested using low brightness on digital devices in the evening to help unwind. As one child explained,

*Sometimes it’s because I’ve been watching for too long*,* and sometimes it’s about what I’m watching. Or it could be how long I watch and what the brightness is like. If it’s too bright*,* I perk up. If it’s not too high*,* it’s more that I don’t have to work so much with my eyes*,* so my eyes relax if I have lower brightness.* (Child 12 years)

These experiences illustrate that digital devices can either support or hinder sleep initiation, depending on individual preferences and usage habits. This highlights the need for personalized approaches to managing digital routines before bedtime.

#### Balancing activities with rest

The children described how balancing activities and rest during the day could promote sleep initiation. For some, resting after school, either by enjoying a moment of peace or taking a nap, was described as a behavioral strategy that helped them recharge and enabled participation in leisure activities later in the day, which was described as a prerequisite for overall well-being. As one child noted,

*You kind of get rested*,* and then it’s like you wake up and it’s all fresh*,* like you’ve slept through the whole night. Anyway—I’m still sleeping really deeply even if it’s the middle of the day. Then I can usually get up again and feel pretty awake. I can go and exercise then and there and do stuff like that.* (Child 12 years)

However, other children described avoiding naps during the day as it worsened their sleep initiation. *”I zone out*,* like I’ll stare at the same thing for maybe five minutes. I close my eyes*,* then open them again*,* because I can’t nap during the day*,* or else I won’t be able to sleep at night.”*(Child 10 years). This variation highlights the importance of individualized routines to support sleep initiation and daily functioning.

The children also noticed differences between weekdays and weekends, with more sleep on weekends leaving them feeling more energized. Balancing rest and activity during the day was linked to improved sleep and overall well-being, with the children describing themselves as happier and more patient after a good night’s sleep.

### Adapting environmental factors

The children with ADHD and sleep problems experienced that adapting environmental factors was essential in facilitating sleep initiation. They described how creating a calming sleep environment and establishing consistent evening routines helped them fall asleep more easily. These routines, largely facilitated by their parents, provided a sense of predictability and security, which significantly enhanced their ability to initiate sleep.

#### Creating a calming sleep environment

The children noted that creating a calming bedroom environment was a beneficial part of their nightly routine to help them unwind and sleep better. A well-organized home environment, characterized by predictable routines and supportive parental involvement, was described as essential for establishing a stable environment that could promote relaxation and prepare them for sleep. The children explained how various factors, such as sound, temperature, cozy pillows, customized bed sizes, the presence of parents, and certain comforting items, played a crucial role in achieving a peaceful state.

*I usually sleep on my stuffed animals*,* and I have my favorite in my hand. Because this particular stuffie is my favorite*,* because it’s kind of small like this. It lies down like this*,* and then it smiles and has its ears up like this. And it’s fluffy and has little beads inside that are a bit hard*,* kind of like a weighted blanket. And you can squeeze it.*(Child, 8 years).

Being tucked into bed, for example, with a weighted blanket, further enhanced their sense of comfort. One child shared, “*When mom and dad leave my bed*,* the weighted blanket hugs me.”* (Child, 6 years).

The children also emphasized the need for just the right amount of light to help them settle and prepare for sleep. As one child explained,

*It’s quite dark in my room*,* so that’s part of why I have trouble sleeping. It gets really dark in there*,* but the problem is if I pull the blinds down*,* it gets so dark that I get scared. But if I pull them up*,* it’s way too bright*,* and then I can’t sleep. I have a little pink lamp*,* though*,* that I sometimes turn on*,* and that makes it just right.* (Child 8 years)

Some preferred cozy lighting for comfort and to feel protected from the dark, while others preferred complete darkness and used a sleep mask. One 10-year-old child shared, “*I have an eye mask*,* so it’s not that hard for me. My eyes get kind of tired when I wear it*,* and I fall asleep pretty easily”.*

Despite their efforts, supported by their parents, to create a peaceful sleep environment, some children still struggled to fall asleep in other settings. Children who alternated between households noted that variations in light, temperature, sound, and bed size, could affect their ability to initiate sleep. Other factors, such as uncomfortable pillows or disruptive siblings or pets, were also mentioned as obstacles to restful sleep. For example, during sleepovers or holidays, they found it more difficult to settle.

*And maybe if I… I’m going to sleep in a tent at a friend’s house today*,* and I get excited when I’m sleeping somewhere new*,* so it’s harder to fall asleep then*,* too.* (Child 10 years)

Creating a calming sleep environment was recognized as essential for adapting environmental factors that support sleep initiation and promote better sleep.

#### Establishing consistent evening routines

The children shared that establishing consistent evening routines helped create a sense of safety and relaxation during sleep initiation. These routines, often supported by active parental involvement and predictable schedules, helped children unwind and prepare for sleep. Common activities included spending time with parents, having a light evening meal, showering, brushing teeth, going to the toilet, or cuddling with comforting items like toys. Some children also found creative activities, such as drawing pictures or painting, helpful to wind down. One child shared,

*We read for ten minutes*,* then mom stays lying there for about three minutes… Then maybe dad comes and stays for a minute… After that*,* I lie down and fall asleep. First*,* I turn over and grab my cuddly toys.* (Child 7 years)

To relax, some children engaged in calming activities, like squeezing a stress ball, while others listened to books or music to ease into sleep. One 10-year-old child explained:

*They actually made me feel sleepier*,* pretty quickly. Especially one book I listened to a lot. It was one of those books with calm words*,* which I think also made me feel tired.* (Child 10 years)

Conversely, children alternating between two households often experienced varying evening routines, which sometimes made it easier for them to initiate sleep in one-parent homes compared to the other. While they occasionally resisted going to bed due to a lack of tiredness, they acknowledged the importance of consistent evening routines in fostering relaxation and a sense of security, which they believed were essential for sleep initiation. The children expressed feeling more secure when their parents were either in the bedroom or nearby as they prepared for sleep. One child shared, *The most important thing is that I have an adult*,* two adults nearby* (Child 9 years).

Establishing consistent evening routines was an important aspect of environmental factors, as it helped the children feel secure and relaxed, ultimately promoting successful sleep initiation.

## Discussion

The primary findings revealed that children with ADHD and sleep problems highlighted the importance of adapting both behavioral and environmental factors to facilitate sleep initiation. While many of these strategies align with existing clinical recommendations, this study adds depth by presenting the children’s own descriptions of how and why such adaptations matter. These adjustments were perceived as important for improving their ability to fall asleep. Key behavioral factors included engaging in physical exercise, managing digital device usage, and balancing active periods with rest. Furthermore, the environmental factors, primarily supported by their parents, involved creating a calming sleep environment and establishing consistent evening routines.

### Adapting behavioral factors

The results show that children’s engagement in physical activities can facilitate sleep initiation, albeit the type and intensity of the activity are important factors. Although research has consistently shown a positive association between physical activity and sleep in children [37, 38], the children’s narratives reveal that the type, intensity, and timing of activity were critical for their ability to relax. Some children found certain activities helpful for sleep initiation, while others experienced increased stimulation, making it harder for them to wind down. These findings underscore the need for a person-centered approach to physical activity, rather than a one-size-fits-all strategy, for children with ADHD. Given that children with ADHD are generally less physically active than their peers [39], it is crucial for parents to encourage participation in physical activities. Research has shown that when parents lead active lifestyles, children are more likely to mirror this behavior, making physical activity easier to adopt [40].

Children in our study noted that physical activity during the day helped them feel more relaxed in the evening. This observation is consistent with research linking high-intensity physical activity [41], and targeted interventions, such as jogging [37], can reduce sleep problems among children with ADHD [37, 41]. Similarity, parents of children with ADHD have reported that physical activity not only improves children’s sleep but also boosts energy, and fosters a positive cycle between exercise and rest [24]. In contrast, the link between physical activity and sleep is less pronounced in healthy children [42, 43].

While some children in our study used digital devices to relaxing before bed, others found screen use overstimulating leading to longer times to fall asleep. This finding is consistent with research showing that screen use can delay sleep initiation [18], reduce sleep duration, impair sleep quality, and increase daytime sleepiness [44, 45]. Despite this, the evidence on this topic remains mixed [46]. Factors such as content type and screen brightness could explain why some children found screens helpful for relaxation, while others found them disruptive [19]. These nuanced findings suggest that the role of screen use in sleep initiation is complex and may vary among children, which is also reflected in previous qualitative research on older children [15, 16, 25].

In addition to physical activity and screen time, balancing rest periods plays a crucial role in sleep initiation. Some children expressed the need to rest after school, to recharge, which allowed them to better engage in leisure activities with their peers. This was also noted by parents, who observed that children with ADHD enjoy and engage more in leisure activities following a good night’s sleep, as they have more energy [24]. On weekends, when the children could sleep longer and wake up refreshed, they reported feeling more energized and better able to participate in leisure activities. This aligns with research that highlights that quality sleep is essential for children with ADHD to regulate their emotions and behavior [10, 24]. Improving sleep outcomes was described as not only facilitating easier sleep initiation but also enhancing a child’s ability to manage daily demands [47], thereby enhancing their overall quality of life. In summary, adapting behavioral factors such as physical activity, screen time, and rest can optimize sleep initiation in children with ADHD and promote well-being. By centering the children’s own accounts, this study provides nuanced insights that extend existing parent- and expert-driven recommendations, highlighting the value of incorporating children’s perspectives to refine and individualize sleep support strategies. These findings correspond with European expert guidelines recommending structured daily routines that balance activity and rest to support sleep initiation [48]. Moreover, recent systematic reviews highlight that behavioral sleep interventions for children with ADHD should be individualized, reflecting the nuances in how children experience and manage rest and activity [49, 50]. Taken together, these results emphasize the importance of integrating children’s voices into both research and clinical practice, ensuring that sleep guidelines and interventions are truly responsive to their everyday lives.

### Adapting environmental factors

The results reveal that creating a calming bedroom atmosphere was crucial for the children to feel at peace and facilitate sleep initiation. Being tucked into bed with a weighted blanket further enhanced their sense of comfort. Although prior recommendations often highlight general principles such as quiet and comfortable sleeping environments [48], the children’s narratives offered concrete examples of the sensory and emotional elements that shaped a sense of relaxation. Previous research indicates that a relaxing bedroom environment can positively impact sleep problems in healthy children [51].

In this study, children emphasized the importance of personalized lighting and temperature settings. Preferences varied widely: some children preferred bright rooms, while others favored darkness, and temperature preferences ranged from cool to warm. While a bedroom that is too warm is known to disrupt sleep [52], this variability suggests the need for an individualized bedroom climate, as also noted by adolescents as a facilitator for good sleep [15, 25]. The results also indicate that surrounding noises were identified as a barrier to sleep initiation. Research suggests continuous background sounds can mask disruptive noises, promoting better sleep [53]. However, the children in this study spoke more about their individual sensory preferences than about general noise recommendations.

An important finding was that children experienced more difficulty initiating sleep outside their well-adapted home environments, emphasizing the importance of a tailored sleep environment, especially for those with ADHD. This reliance on consistency and structure is well-documented as a coping strategy for sleep problems in children with ADHD [14]. An intriguing finding was that children who alternated between the households of their parents faced challenges due to differing evening routines, limits, and household environments [19]. Inconsistent parental work schedules may also have contributed [54]. The children expressed feeling less able to control their sleep environment in unfamiliar settings, sometimes leading to avoidance of sleepovers or travel due to anxiety about disrupted routines [22].

Children in this study also described how specific sensory elements, such as the feel of bedding, the presence of favorite objects, and adjustable lighting, were central to creating a calming sleep environment. These individualized preferences often went beyond the more general recommendations in clinical guidelines and parent-reported studies, which typically emphasize the importance of a quiet, dark, and comfortable bedroom and address issues like bedtime resistance [23, 48]. The children’s accounts highlight concrete examples of how such environmental recommendations can be tailored and implemented in daily life. Systematic reviews have similarly emphasized the importance of addressing unique sensory sensitivities when designing interventions for children with ADHD [49]. These insights illustrate how children’s voices can enrich and specify existing recommendations, supporting the development of more responsive and individualized sleep support strategies.

The findings underscore the importance of establishing consistent evening routines, which helped the children feel safe and relaxed, thereby facilitating sleep initiation. These routines often involved spending time with parents, emphasizing the role of parental involvement and structured routines. This aligns with current European guidelines, which recommend predictable evening routines to support sleep initiation in children [48]. Prior research also highlights the need for parents to maintain consistent and nurturing evening routines [14, 55]. Recent systematic reviews further emphasize the role of tailoring behavioral sleep interventions, including predictable evening routines, for supporting sleep in children with ADHD (Becker et al., 2022; Larsson et al., 2023). In this study, consistent routines were experienced as beneficial even when children resisted bedtime, as parental involvement provided a sense of security. The children described not only the sequence of activities but also the sensory and emotional elements, calming activities, comforting objects, and the reassuring presence of a parent, that made these routines effective. These nuanced accounts add depth to existing knowledge, which often focuses on general routine adherence and bedtime resistance [14, 48, 55]. Involving children in shaping their evening routines and bedroom environments has been shown to enhance their sleep experience [15, 25]. By foregrounding children’s voices, this study refines existing recommendations, highlighting the role of personalized and responsive approaches in supporting sleep initiation for children with ADHD.

### Implications for interventions

While the findings align with existing recommendations for supporting sleep in children with ADHD, this study highlights the added value of incorporating children’s own feedback and preferences more explicitly when designing and implementing interventions. The results emphasize the need for a flexible, person‑centered approach grounded in the lived experience of the child. Given the qualitative and exploratory nature of this study, it is important to avoid causal claims. Instead, the findings should be viewed as providing valuable insights into how children perceive facilitators and barriers to initiating sleep, insights that can help adapt and refine existing interventions to better fit their everyday context and needs.

### Methodological considerations

Trustworthiness in qualitative research is commonly evaluated through four criteria: credibility, dependability, confirmability, and transferability [28]. Credibility in this study was enhanced by including 21 children with direct experiences of ADHD and sleep problems, thereby offering a wide range of authentic insights. The use of open-ended questions allowed the children to express their views in their own words, further enhancing the study’s credibility. In addition, data collection was guided by the principle of saturation, whereby interviews continued until no new relevant information emerged from the data. This approach strengthened the credibility of the findings, suggesting that the identified categories reflect recurring and meaningful aspects of the children’s experiences. Dependability was strengthened by employing an interview guide across all interviews, conducted via Microsoft Teams or Zoom, due to the COVID-19 pandemic or face-to-face, which introduced some variability. While remote interviewing via videoconferencing platforms presents certain advantages and might be preferable in some contexts [56], the variability also presents a potential limitation. Dependability was further addressed by iterative discussions among a multidisciplinary team, whose combined methodological and subject expertise helped in reaching a consensus during analysis. Reflexivity was an important part of the research process, as all five researchers are parents with prior experience working with children in research. Throughout the analysis and interpretation, ongoing reflexive discussions were held to critically examine the researchers’ preunderstandings and to ensure that the children’s voices were prioritized.

Confirmability was strengthened by multiple quotations from different children in each subcategory, which captured their voices and experiences of sleep problems while illustrating the data and accurately conveying their perspectives. Purposive sampling enhanced transferability and ensured a diverse representation of boys and girls across ages 6–12 years, enriching the perspectives. However, the fact that all participants were recruited from a single setting might limit the transferability of the findings. With respect to transferability, it is also important to note that most participating families had relatively stable living conditions, with the majority of parents working either full- or part-time, and all having at least upper secondary education. Additionally, nearly all families were born in Sweden, and most parents lived in partnered relationships. These characteristics suggest that the families were not living in social exclusion or facing severe socioeconomic challenges, which may influence the transferability of the findings to contexts with different family constellations, cultural backgrounds, or economic situations. An additional limitation is that this study did not specifically explore sensory processing dysfunctions or restless legs syndrome as potential comorbidities affecting sleep initiation and quality in children with ADHD [57, 58]. Previous research highlights discomfort- and pain-related movements as significant contributors to difficulties falling asleep and non-restorative sleep [11, 59]. Including these aspects in the interviews could have provided a deeper understanding of children’s experiences with sleep initiation and further strengthened the clinical relevance and transferability of the findings. Future research should consider incorporating targeted inquiries or assessments of these factors to clarify their role in sleep challenges within this population. Despite this limitation, the study’s rich and nuanced results offer valuable insights that inform understanding in similar contexts.

## Conclusions

This study underscores the importance of tailoring behavioral and environmental factors to support sleep initiation in children with ADHD and sleep problems. The findings suggest that supporting children to engage in structured physical activity, regulating digital device use, and maintaining a balance between activity and rest may be important for sleep facilitation, according to children’s own perspectives. Furthermore, the study highlights the pivotal role of parental support in implementing environmental adjustments, such as creating a calming sleep environment and establishing consistent routines. These findings offer valuable knowledge into how children with ADHD and sleep problems experience and describe sleep facilitation, which may inform future research and practice in the field. Rather than implying causation or direct intervention strategies, these exploratory insights can help shape approaches that better incorporate children’s feedback and preferences.

## Data Availability

The datasets generated during and/or analyzed during the current study are not publicly available due to legal/ethical reasons but are available from the corresponding author on reasonable request.
